# Intestinal adhesion due to previous uterine surgery as a risk factor for delayed diagnosis of uterine rupture: a case report

**DOI:** 10.1186/1752-1947-5-523

**Published:** 2011-10-23

**Authors:** Tomoyuki Kuwata, Shigeki Matsubara, Rie Usui, Shin-ichiro Uchida, Naohiro Sata, Mitsuaki Suzuki

**Affiliations:** 1 Department of Obstetrics and Gynecology, Jichi Medical University, Tochigi, Japan; 2 Jichi Perinatal Education Center, Jichi Medical University, Tochigi, Japan; 3 Department of Surgery, Jichi Medical University, Tochigi, Japan

## Abstract

**Introduction:**

Uterine rupture is a life-threatening condition both to mothers and fetuses. Its early diagnosis and treatment may save their lives. Previous myomectomy is a high risk factor for uterine rupture. Intestinal adhesion due to previous myomectomy may also prevent early diagnosis of uterine rupture.

**Case presentation:**

A 38-year-old primiparous non-laboring Japanese woman with a history of myomectomy was admitted in her 34^th ^week due to lower abdominal pain. Although the pain was slight and her vital signs were stable, computed tomography revealed massive fluid collection in her abdominal cavity, which led us to perform a laparotomy. Uterine rupture had occurred at the site of the previous myomectomy; however, the small intestine was adhered tightly to the rupture, thus masking it. The baby was delivered through a low uterine segment transverse incision. The ruptured uterine wall was reconstructed.

**Conclusion:**

Intestinal adhesion due to a prior myomectomy occluded a uterine rupture, possibly masking its symptoms and signs, which may have prevented early diagnosis.

## Introduction

Uterine rupture is a life-threatening condition both to mothers and fetuses [1]. Early diagnosis of uterine rupture and awareness of its risk factors are clinically important. Previous uterine surgery, such as Cesarean section, myomectomy or adenomyomectomy, is a risk factor [2-4]. Here, we report a prelabor uterine rupture at a previous myoma enucleation site, in which intestinal adhesion to the ruptured site occluded the rupture, possibly preventing early diagnosis.

## Case presentation

A 38-year-old Japanese primiparous woman with a history of myomectomy four years previously complained of lower abdominal pain in her 34^th ^week. This was her second pregnancy with spontaneous conception, with her first pregnancy resulting in spontaneous abortion at six weeks one year earlier. Her past history was unremarkable except for a history of myomectomy, which was performed for infertility (secondary sterility) for approximately three years. Myomectomy was performed under laparotomy, and eight intramural myomas in the uterine body were enucleated. The largest one (40 × 50 mm) existed in the anterior uterine body, which was enucleated with vertical incision. The enucleation sites had been reconstructed using routine two-layered sutures. Her uterine cavity was not entered. Surgery took 100 minutes and the total amount of hemorrhage was 550 mL, requiring no transfusion. She had had an uneventful postsurgery course without fever.

A physical examination revealed tenderness in the middle of her lower abdomen without guarding. She showed no vaginal bleeding. Her blood pressure was 106/64 mmHg, pulse rate 81 beats/min, white blood cell count 9.2 × 10^9^/L, and hemoglobin 9.4 g/dL. She had no postural hypotension. Cardiotocography (CTG) indicated a reassuring pattern with weak uterine contractions once per hour. A vaginal and abdominal ultrasound revealed no fluid retention in Pouch of Douglas and no apparent uterine rupture; although no detailed observation of uterine wall continuity was made. Slight abdominal pain continued with stable vital signs and unremarkable laboratory data.

**Figure 1:**
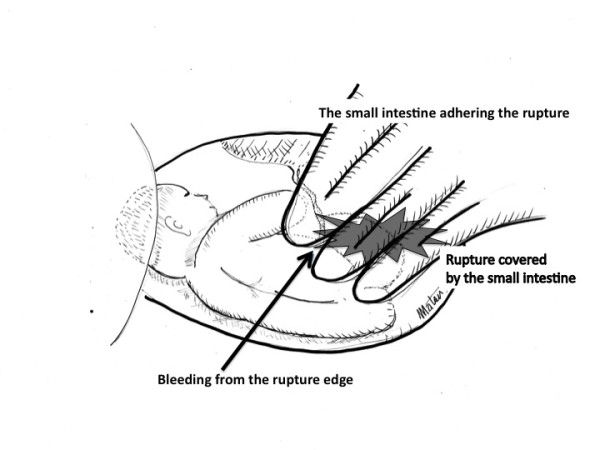
**Schematic diagram of the laparotomy findings**. The uterine rupture was not initially discernable. Bleeding was observed from the rupture edge (arrow). Her small intestine tightly adhered the anterior uterine wall. After separating the small intestine, uterine rupture became evident; her small intestine covered and occluded the uterine rupture. Amniotic membrane beneath the rupture site remained intact.

Six hours later, she complained of upper abdominal pain. Computed tomography (CT) revealed fluid accumulation around her liver. Surgeons diagnosed this condition as probable perforated viscus or at least acute abdomen requiring laparotomy. CTG subsequently indicated recurrent late deceleration, requiring an emergent Cesarean section. Laparotomy revealed that her small intestine tightly covered the anterior uterine wall, with bleeding observed from the edge of the intestinal covering (Figure 1, arrow). After separating her small intestine, it became evident that the anterior uterine wall, corresponding to the previous myomectomy site, was ruptured, with her small intestine tightly adhering to the ruptured site and thus nearly completely occluding the rupture (Figure 1). Pouch of Douglas was not entered due to adhesion. A low segmental transverse incision yielded a 2304-g female baby with Apgar scores of 2, 4 and 7 at one, five and ten minutes, respectively. Her small intestine was freed from the rupture site. The 5-cm longitudinal rupture of the anterior uterine wall was reconstructed. Her total blood loss during the surgery was 3750 mL, and she received a transfusion with 2000 mL hemoperitoneum, 1200 mL allogeneic blood and six units of fresh frozen plasma. The mother and baby had an uneventful course without sequelae.

## Discussion

In our reported case, intestinal adhesion covered and occluded a uterine rupture, which may have masked the symptoms typical to uterine rupture, possibly preventing early diagnosis. To the best of our knowledge, no previous report describes this phenomenon. The course of our patient was considered to be as follows.

The rupture may have occurred around or before the time of admission; however, the small intestine covering the ruptured site may have prevented acute massive bleeding, which may be why vital signs and laboratory data were stable. Covering by the small intestine may have also prevented amniotic rupture or amniotic cavity protrusion, which may explain the initial absence of a fetal heart rate pattern indicative of cord troubles. The Pouch of Douglas was closed, possibly due to the previous laparotomy, prohibiting blood retention. The ruptured site bled continuously with the blood accumulating around the liver, causing upper abdominal pain. The rupture may have increased, causing fetal heart rate pattern abnormalities.

Kurdoglu *et al. *[5] reported a uterine rupture case: the rupture was considered to have occurred due to assisted fundal pressure at delivery. The diagnosis was made 32 hours postpartum; postural hypotension was the sign that attracted the physicians' attention, leading to the diagnosis. The present case did not show postural hypotension. Our patient remained lying in bed with little postural change, which may explain why she showed no postural hypotension.

Considering that the adhesion was very tight and that adhesion to the myomectomy site is a frequently observed phenomenon, the intestinal adhesion to the rupture may have been present well before, and not after, the rupture. Thus, uterine rupture occurred in the enucleation scar site on which the small intestine tightly adhered.

A recent article also described uterine rupture occluded by 'fetal legs'. Blihovde *et al. *[6] described a prelabor primiparous uterine rupture at the 32^nd ^week of gestation, with the ruptured site being occluded by the fetal legs. She had abdominal pain but without vaginal bleeding, hemodynamical instability or fetal compromise. The physicians suspected appendicitis; however, CT revealed the uterine rupture occluded by the protruding fetal legs from the ruptured site, which was confirmed by laparotomy. The fetal legs, protruding through the rupture and occluding it, masked the symptoms and signs of the rupture, delaying the diagnosis.

The article by Blihovde *et al. *[6] concluded, 'clinicians should consider the diagnosis of uterine rupture when a patient presents with abdominal pain, even without evidence of hypovolemia, vaginal bleeding, contractions, or fetal compromise'. This statement is supported by the present case. While intestinal adhesion covered and delayed the diagnosis of the rupture in our case, fetal legs had covered, and thus masked, the rupture in their case.

Previous uterine surgery is a well-known risk factor for uterine rupture even before labor, as previously described [2-4]. Previous myomectomy, inducing a tight intestinal adhesion at the site, may mask the symptoms and signs of a rupture. We cannot exclude the possibility that intestinal adhesion might have been a coincidental phenomenon. However, two patients were reported in whom gastric peptic ulcer perforation was covered by the adhesion of the abdominal wall to the perforation sites, which masked typical symptoms and signs of gastric ulcer perforation [7]. We note the similarity between these two cases and the present case. Although it could not be determined whether intestinal adhesion delayed the diagnosis of rupture, we must consider this possibility in pregnant women after myomectomy. Moreover, intestinal adhesion occurs not only after myomectomy but also after any other abdominal surgeries, and thus we must be cautious about this possibility in dealing with pregnant women after abdominal surgery.

## Conclusions

Myomectomy may be a risk factor for uterine rupture, not only causing the rupture but also masking it and thus preventing its early diagnosis.

## Abbreviations

CT: computed tomography; CTG: cardiotocography.

## Consent

Written informed consent was obtained from the patient for publication of this case report and any accompanying images. A copy of the written consent is available for review by the Editor-in-Chief of this journal.

## Competing interests

The authors declare that they have no competing interests.

## Authors' contributions

TK, SM, SU and RU diagnosed, investigated, followed-up and managed the patient, and determined the medical significance. SM and TK wrote the manuscript. TK and NS revised the manuscript. NS and MS provided important suggestions regarding medical content. All authors read and approved the final manuscript.
